# AMPK activation enhances osteoblast differentiation on a titanium disc via autophagy

**DOI:** 10.1186/s40729-024-00525-2

**Published:** 2024-01-29

**Authors:** Kei Egashira, Hiroshi Kajiya, Takashi Tsutsumi, Yusuke Taniguchi, Kae Kakura, Jun Ohno, Hirofumi Kido

**Affiliations:** 1 Section of Oral Implantology, Department of Oral Rehabilitation, Fukuoka Dental College, Fukuoka, Japan; 2 Oral Medicine Research Center, Fukuoka Dental College, Fukuoka, Japan; 3 Department of Physiological Science and Molecular Biology, Fukuoka Dental College, Fukuoka, 814-0193 Japan; 4 Department of General Dentistry, Fukuoka Dental College, Fukuoka, Japan

**Keywords:** Titanium disc, AMP-activated protein kinase, Osteoblast differentiation, Autophagy, Osseointegration

## Abstract

**Purpose:**

The acquisition of osseointegration during implant therapy is slower and poorer in patients with diabetes compared with healthy persons. The serum concentration of adiponectin in patients with type II diabetes is lower than that of healthy persons via the suppression of AMP-activated protein kinase (AMPK). Therefore, we hypothesized that the AMPK activation enhances bone formation around implants, resulting in the improved acquisition of osseointegration. The purpose of this study was to evaluate the impact of AMPK activation on osteoblast differentiation and its mechanism of downstream signaling on titanium disc (Ti).

**Methods:**

Confluent mouse pre-osteoblasts (MC3T3-E1) cells (1 × 10^5^ cells/well) were cultured with BMP-2 for osteoblast differentiation, in the presence or absence AICAR, an AMPK activator. We examined the effects of AMPK activation on osteoblast differentiation and the underlying mechanism on a Ti using a CCK8 assay, a luciferase assay, quantitative RT-PCR, and western blotting.

**Results:**

Although the proliferation rate of osteoblasts was not different between a Ti and a tissue culture polystyrene dish, the addition of AICAR, AMPK activator slightly enhanced osteoblast proliferation on the Ti. AICAR enhanced the BMP-2-dependent transcriptional activity on the Ti, leading to upregulation in the expression of osteogenesis-associated molecules. AICAR simultaneously upregulated the expression of autophagy-associated molecules on the Ti, especially LC3-II. AdipoRon, an adiponectin receptor type1/type2 activator activated AMPK, and upregulated osteogenesis-associated molecules on Ti.

**Conclusions:**

AMPK activation enhances osteoblast differentiation on a Ti via autophagy, suggesting that it promotes the acquisition of osseointegration during implant therapy.

**Supplementary Information:**

The online version contains supplementary material available at 10.1186/s40729-024-00525-2.

## Background

AMPK has been reported to be a conserved sensor of cell energy conditions [1]. AMPK has previously been reported to be activated by metabolic stresses and to switch on catabolic pathways while switching off biosynthetic pathways to maintain the intracellular energy equilibrium [2]. AMPK activation promotes cell viability and mitochondrial biogenesis and fission, as well as the expression of antioxidant enzymes [3]. There are some reports that AMPK has been reported to stimulate osteoblast differentiation and mineralization in pre-osteoblast cell lines on tissue culture polystyrene dishes (TCPD) using osteogenesis induced medium (OIM) [4, 5]. Furthermore, AICAR, an AMPK activator stimulated the autophagy signaling pathway in osteoblast lineages including mesenchymal stem/stromal cells on TCPD [6, 7]. We recently reported that AICAR upregulated osteoblastic mineralization on titanium disc (Ti) [8], but not TCPD.

However, most of these reports were obtained from osteoblasts cultured on TCPD using osteogenesis induced medium (OIM), and few have been investigated those of titanium. Furthermore, no reports have verified autophagy-regulated osteoblast differentiation on titanium, but not on TCPD.

Adiponectin is a fat-derived adipokine that plays an important role in controlling the balance of energy metabolism at a systematic level via AMPK activation [9, 10]. The concentration of adiponectin in the serum is significantly reduced in patients with type 2 diabetes, resulting in diabetes-induced bone loss [11]. Therefore, AMPK, adiponectin downstream signaling has been proposed to be a potential therapeutic target to promote osseointegration in bone-to-implant contact and osseointegration, especially in patients with type 2 diabetes [12].

In the present experiments, we hypothesize that the addition of AMPK activator enhances osteoblast differentiation in the around peri-implant bone, leading to the promotion of earlier and stronger bone-to-implant contact and osseointegration, even though diabetes. We examined the effects of AMPK activation on the expression of osteoblast differentiation-associated molecules on a Ti in mouse pre-osteoblasts, as well as its underlying mechanism.

The purpose of this study was to evaluate the impact of AMPK activation on osteoblast differentiation and its mechanism of downstream signaling on titanium discs.

## Materials and methods

### Ti preparation

The titanium discs (10.0 × 10.0 × 1.0 mm; NANTOH Co., Ltd., Shizuoka, Japan) and *φ*55 mm × 1 mm disc (TIG Co., Ltd, Osaka, Japan) consisted of JIS Class 4 machine polished discs with a surface roughness (Sa: 0.176.6 ± 0.054 mm).

### Surface topography of Ti

**Fig. 1:**
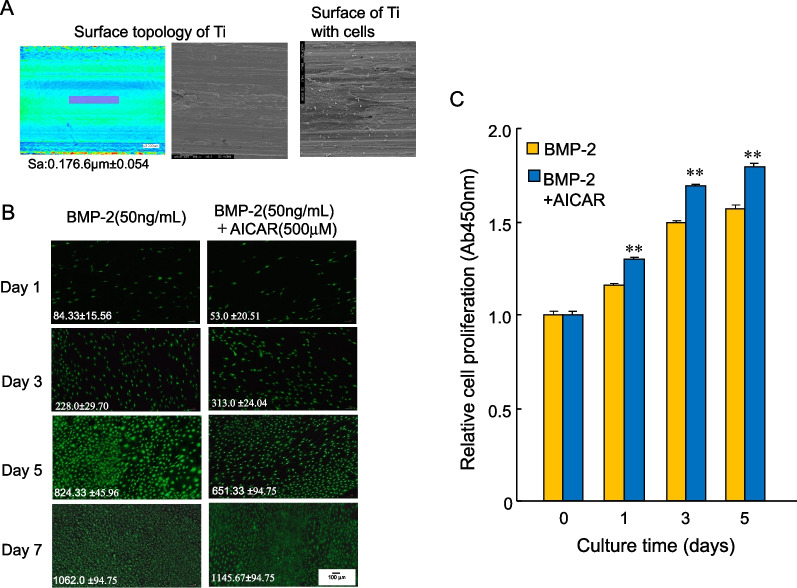
AICAR slightly upregulated the BMP-2-induced proliferation rate in osteoblasts on a titanium disc. **A** Characterization of material surface of titanium. Surface topology of Ti using laser fiber scanning and those in without or with MC3T3-E1 cells cultured after 24 h using scanning electron microscope. **B** MC3T3-E1 cells were treated with BMP-2 (50 ng/mL) in the presence or absence of AICAR (500 μM) and stained on a titanium disc using calcein–AM. The number inserted at the bottom left of each photo indicates the mean from the positive cells on three Ti discs (mean ± SEM). **C** Cells treated with BMP-2 (50 ng/mL) were cultured with or without AICAR (500 μM) on the titanium disc, and the rate of cell proliferation as determined using a CCK-8 assay. The bar graphs show the mean from five culture wells (mean ± SEM). **Indicates *P* < 0.01 vs. treatment with BMP-2 alone

Titanium surface roughness was evaluated parameter the arithmetic mean height of the surface (Sa) using a fiber laser (MD-F3000, Keyence, Osaka, Japan: Fig. 1A), with a 50 × 50 mm filter in three different areas on each sample. Characterization of material surface and condition of cells on titanium. MC3T3E1 cells cultured on titanium disc at 24 h. Cells were fixed with 10% formalin, dehydrated in grated concentrations of ethanol, and dried by liquefied CO_2_ using a critical point dryer. Samples were sputtered with gold for 2 min and observed by scanning electron microscope (JSM-6330F, JEOL, Tokyo, Japan).

### Cell culture

Mouse pre-osteoblast cells (MC3T3-E1; subclone 14) were obtained from the American Type Culture Collection (Manassas, Virginia, USA). The cells were cultured in minimum essential medium-α (MEM-α; Fujifilm Wako, Tokyo, Japan) supplemented with 10% fetal bovine serum (Sigma–Aldrich, St. Louis, MO, USA) and penicillin–streptomycin (Fujifilm Wako, Tokyo, Japan) until the cells reached a confluence of 70%. After reaching sub-confluence, the cells (1.0 × 10^5^ cell/mL) were culture on titanium discs with BMP − 2 (25–50 ng/mL, Pepro Tech. Inc., NJ, USA) or an osteoblast-inducing medium (OIM; including glycerophosphate and ascorbic acid), for osteoblast differentiation. In parallel experiments, cells were incubated with BMP-2 plus AICAR (500 μM). In some experiments, to clarify effect of AICIR on osteoblast differentiation via autophagy 3-MA (3-methhyladnine; Fujifilm Wako, Tokyo, Japan), a PI3Kinase inhibitor were applied to the cells for 72 h. Furthermore, to investigate whether adiponectin stimulates on osteoblast differentiation via AMPK activation AdipoRon (Enzo Life Sciences, NY, USA) were applied to the cells for 72 h.

### CCK-8 proliferation assay

Cellular proliferation was measured using a counting Kit-8 assay (CCK-8; Dojindo Laboratories, Kumamoto, Japan). Briefly, the cells were seeded in 24-well plates at a density of 1.0 × 10^4^ cells/mL in 500 μL of MEM-α. After each cultivation, the CCK-8 reagent (10 μL) was added to each well and the plates were incubated at 37 °C for 1 h. After the collection of conditional culture medium (100 μL) from each well, absorbance was read at 450 nm using a microplate reader (Mode 680; Bio-Rad). Culture solution containing the CCK-8 reagent but without cells was used as a blank control.

### Calcein staining

Cells (10 × 10^4^ cells/mL) were plated on a titanium disc inserted in a 24-well plate (500 μL/well). After cultivation for 7 days, cells on the titanium disc were stained with calcein–AM solution (1 µL/mL, DOJINDO). The number of cells on the titanium disc was observed under a fluorescence microscope.

### Transcriptional activity assay

For promoter assays, the Id promoter was cloned into the pGL4 basic luciferase plasmid (Promega; Madison, WI, USA), and the resulting construct was transfected into MC3T3-E1 cells using Lipofectamine 3000 (Thermo Fisher, CA, USA). Luciferase activity was measured using a luciferase reporter assay system (Promega). These cells were cotransfected with the pCMV-galactosidase reporter plasmid (Promega) and the transfection activity was normalized by measuring the galactosidase activity.

### RNA isolation and quantitative reverse transcription polymerase chain reaction

Total RNA was extracted from the cells using the TRIzol reagent. First-strand complementary deoxyribonucleic acid (cDNA) was synthesized from 3 µg of total RNA using ReverTra Ace reverse transcriptase according to the manufacturer’s instructions (TOYOBO CO., LTD, Osaka, Japan). To detect mRNA expression, we selected specific primers based on the nucleotide sequence of the resultant cDNA (Additional File 1: Table S1). The relative messenger RNA (mRNA) expression was normalized as the ratio of the expression level of the targeted mRNAs to that of β-actin.

### Western blot analysis

Cells were lysed in TNT buffer [0.1 M Tris–HCl (pH, 7.5), 0.15 M NaCl, 0.05% Tween-20; Roche, Basel, Switzerland]. The protein content was measured using a protein assay kit (Thermo Fisher, CA, USA). Subsequently, 20 µg of each protein were subjected to 12.5% sodium dodecyl sulfate polyacrylamide gel electrophoresis, and the separated proteins were transferred electrophoretically onto a polyvinylidene fluoride membrane at 80 V and 4 °C for 1.5 h. The membrane was incubated with antibodies against Runx2, Osterix/Sp7 (OSX; Abcam, Cambridge, UK), p-Smad1/5/9, Smad1, P-AMPK, AMPK, Beclin-1, LC3B, p-ULK1, ULK-1, BNIP-3 (Cell Signaling Technology, Tokyo, Japan), and β-actin (Sigma–Aldrich Co.) diluted (1:1000) in 5% BSA and Tris-buffered saline with Tween-20 (TBST; 10 mM Tris–HCl, 50 mM NaCl, 0.25% Tween-20) plus 0.01% azide overnight at 4 °C. The blots were washed in TBST and incubated for 1 h with horseradish peroxidase-conjugated anti-rabbit or anti-mouse immunoglobulin-G secondary antibodies. The antibodies were diluted (1:2000) in 5% skimmed milk in TBST and were developed using an enhanced chemiluminescent system (GE Healthcare, Tokyo, Japan).

### Statistical analyses

Data are expressed as the mean ± standard error of the mean. Differences were analyzed by one-way analysis of variance and Scheffe’s multiple comparison test. *P* values of < 0.05 were considered significant.

## Results

### AICAR slightly upregulated the proliferation rate of osteoblasts on a titanium disc (Ti)

To clarify the attachment and viability of MC3T3-E1 cells on a titanium disc (Ti), we first examined the cellular morphology and proliferation on Ti using calcein staining and a CCK-8 proliferation assay (Fig. 1B). MC3T3-E1 cells were less attached on Ti compared with TCPD at day 0. However, BMP-2 (50 ng/mL) enhanced the proliferation of the cells on Ti in a time-dependent manner (Fig. 1C). In turn, BMP-2 (50 ng/mL) in the presence of AICAR (500 μM), which is an AMPK activator, slightly upregulated cell proliferation on Ti in a time-dependent manner compared with BMP-2-induced proliferation (Fig. 1C). The number of proliferating cells in the presence of AICAR remained constant over 5 days or was slightly decreased from day 5 after BMP-2 treatment, resulting in a small effect on proliferation in the presence or absence of AICAR.

### AICAR enhanced BMP-2-dependent transcriptional activity on Ti with expression of osteoblast differentiation-related molecules

**Fig. 2:**
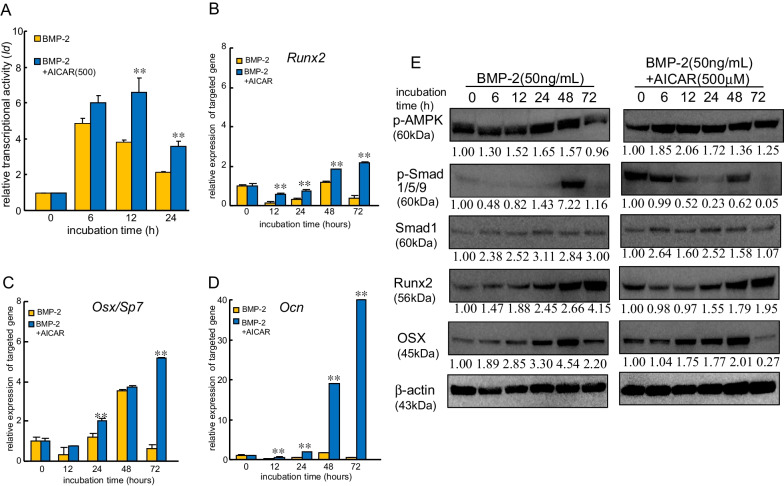
AICAR enhanced the BMP-2-dependent transcriptional activity on Ti via the expression of osteoblast differentiation-related molecules. **A** *Id* promoter site was fused to luciferase and transfected into MC3T3-E1 cells using Lipofectamine 3000. The cells were treated with BMP-2 (50 ng/mL) with or without AICAR (500 μM) for the indicated times during culture, and luciferase activity was assessed as a measure of *Id* transcriptional activity. The data are the mean from four culture wells (mean ± SEM). **Indicates *P* < 0.01 vs. treatment with BMP-2 alone. **B**–**D** MC3T3-E1 cells were incubated with BMP-2 in the presence or absence of AICAR (500 μM). Expression of the osteoblast differentiation-related genes *Runx2* (Runx2), Osx/*Sp7* (OSX/Sp7), and *Ocn* (Osteocalcin) in MC3T3-E1 cells after incubation with BMP-2 (50 ng/mL). Samples were analyzed by quantitative reverse transcription polymerase chain reaction and were normalized to the ß-actin mRNA. The data are the mean from six culture wells (mean ± SEM). **Indicates *P* < 0.01 vs. treatment with BMP-2 alone. **E** Cells were treated with BMP-2 (50 ng/mL) in the presence or absence of AICAR (500 μM). Western blotting was conducted using targeted and ß-actin antibodies. Similar results were obtained in four independent experiments

To clarify whether AICAR regulated osteoblast differentiation in MC3T3-E1 cells on Ti, we examined the impact of AICAR on transcriptional activity using a luciferase reporter assay. BMP-2 transiently and greatly increased the transcriptional activity of *Id*, a BMP-2 downstream signaling gene, in MC3T3-E1 cells on Ti (Fig. 2A). The increased transcriptional activity of *Id* reached a peak at 6 h and declined at 24 h after BMP-2 treatment. Furthermore, the addition of AICAR continuously increased the transcriptional activity, with a peak observed at 12 h in cells on Ti. Furthermore, BMP-2 also induced the expression of the *Runx2*, *OSX/Sp7*, *and Ocn* genes, which are osteoblast differentiation markers, in a time-dependent manner (Fig. 2B–D). Moreover, AICAR addition upregulated the expression of these genes in a time-dependent manner. The increase in the expression of targeted proteins in the presence or absence of AICAR was further confirmed using a western blot analysis (Fig. 2E; Additional File 1: Fig. S1), which yielded similar results, showing that AICAR administration for more than 24 h upregulated the expression of Runx2, OSX/Sp7, and p-AMPK in MC3T3-E1 cells compared with treatment with BMP-2 alone. Similar results were obtained regarding the expression of OIM-induced proteins in MC3T3-E1 cells on Ti using western blot analyses (Additional file 1: Fig. S2). On contrast, AICAR monotreatment slightly upregulated the expression of osteoblast differentiation-related molecules compared with AICAR in presence of OIM (Additional File 1: Fig. S2).

### AICAR simultaneously enhanced the expression of BMP-2-induced autophagy-associated molecules in MC3T3-E1 cells on Ti

**Fig. 3:**
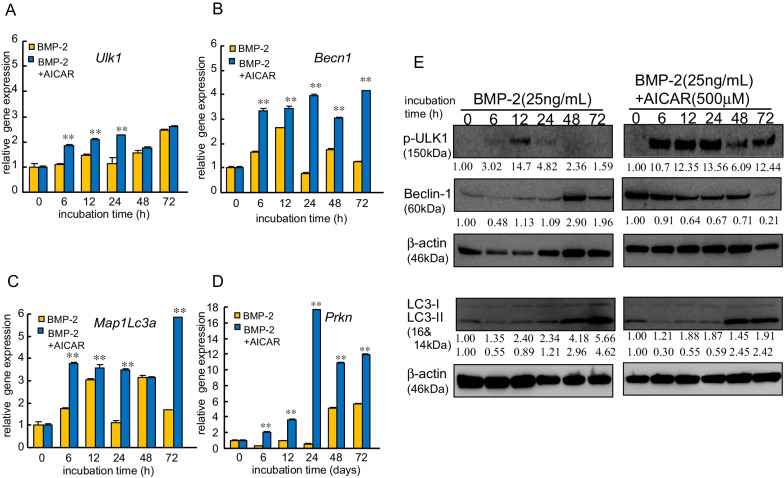
AICAR simultaneously enhanced the expression of BMP-2-induced autophagy-associated molecules, especially LC3B-II, in MC3T3-E1 cells on Ti. **A**–**D** MC3T3-E1 cells were incubated with BMP-2 in the presence or absence of AICAR (500 μM). Expression of the autophagy-related genes *Ulk1* (ULK1), *Becn1* (Beclin-1), *MapLc3a* (LC3-I), and *Pakn* (Parkin) in MC3T3-E1 cells after incubation with BMP-2 (25 ng/mL). The samples were analyzed by quantitative reverse transcription polymerase chain reaction and were normalized to the ß-actin mRNA. The data are the mean from six culture wells (mean ± SEM). **Indicates *P* < 0.01 vs. treatment with BMP-2 alone. **E** Cells were treated with BMP-2 (25 ng/mL) in the presence or absence of AICAR (500 μM). Western blotting was conducted using targeted and ß-actin antibodies. Similar results were obtained in three independent experiments

Osteoblast differentiation has been reported (including our studies) to be regulated by autophagy [13, 14]. To clarify whether AMPK activation enhances osteoblast differentiation in MC3T3-E1 cells on Ti, we examined the effects of AICAR on the osteogenesis of MC3T3-E1 cells on Ti treated with BMP-2. BMP-2 induced the expression of autophagy-associated genes, such as *Ulk1*, *Becn1*, *Map1Lc-3*, and *Parkin*, in MC3T3-E1 cells on Ti in a time-dependent manner, with AICAR addition upregulating their expression (Fig. 3A–D). Similar results were obtained regarding the expression of their target proteins, with BMP-2 gradually increasing the conversion of LC3-I to autophagosome-associated LC3-II in MC3T3-E1 cells on Ti (Fig. 3E; Additional File 1: Fig. S3). Furthermore, AICAR addition for more than 12 h upregulated the expression of their autophagy-associated molecules, together with an increase in the expression of phosphorylated AMPK, especially LC3-II, in MC3T3-E1 cells on Ti. The conversion changes in LC3-II were correlated with the increase in Beclin-1, which is an autophagy marker, in MC3T3-E1 cells. Similar results were obtained regarding the expression of OIM-induced proteins in MC3T3-E1 cells on Ti (Additional file 1: Fig. S2E). On contrast, AICAR monotreatment slightly upregulated the expression of autophagy-related molecules compared with AICAR in presence of OIM (Additional File 1: Fig. S4).

**Fig. 4:**
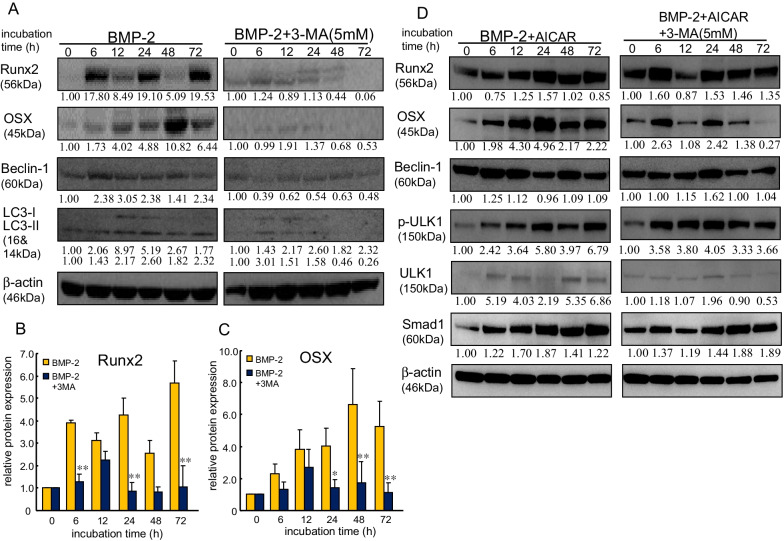
3-MA suppressed BMP-2-induced osteoblast differentiation molecules and autophagy-related molecules. **A **BMP-2 (25 ng/mL)-induced osteoblast differentiation molecules were mediated by autophagy signaling. The cells were incubated with BMP-2 in the presence or absence of 3-MA (5 mM), which is a PI3K inhibitor. **B**, **C **The bar graphs show the mean from five samples (mean ± SEM). * and **Indicate *P* < 0.05 and *P* < 0.01, respectively, vs. treatment with BMP-2 alone. **D** Cells were treated with BMP-2 (25 ng/mL) plus AICAR (500 μM) in the presence or absence of 3-MA (5 mM). Western blotting was conducted using targeted and ß-actin antibodies. Similar results were obtained in three independent experiments

To confirm whether the autophagy-associated molecules regulated osteogenesis, we investigated the inhibitory effect of 3-methhyladnine (3-MA), which is an autophagy inhibitor, on the upregulation of osteoblast differentiation (Fig. 4A; Additional File 1: Fig. S5).

Although autophagy was assessed in the early and late stages of the process, we examined whether autophagy participated in osteoblast differentiation in the early stage using 3-MA, which is an inhibitor of the early stage. Treatment with 3-MA suppressed the expression of Beclin-1 and p-ULK1 in MC3T3-E1 cells on Ti. This treatment simultaneously suppressed osteoblast differentiation markers, such as Runx2 and OSX (Fig. 4B and C). Furthermore, AICAR addition attenuated the suppression of the expression of Runx2 and OSX in MC3T3-E1 cells on Ti, indicating that osteoblast differentiation was regulated by the activation of autophagy, especially in the early stage (Fig. 4D; Additional File 1: Fig. S6).

### AdipoRon, an adiponectin receptor activator, also upregulated osteogenesis-associated molecules

**Fig. 5:**
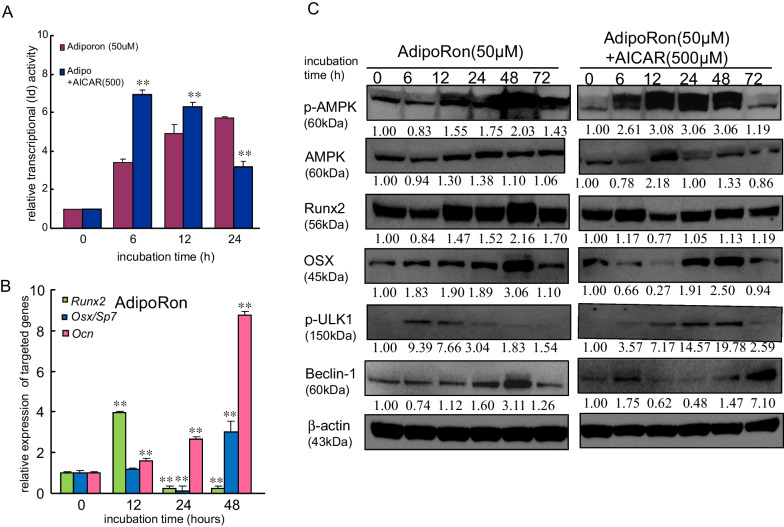
Adiponectin enhanced osteoblast differentiation via the AMPK pathway. **A** *Id* promoter site was fused to luciferase and transfected into MC3T3-E1 cells using Lipofectamine 3000. The cells were treated with AdipoRon (50 μM) with or without AICAR (500 μM) for the indicated times during culture, and luciferase activity was assessed as a measure of *Id* transcriptional activity. The data are the mean from four culture wells (mean ± SEM). **Indicates *P* < 0.01 vs. treatment with AdipoRon alone. **B** MC3T3-E1 cells were incubated with AdipoRon. Expression of the osteoblast differentiation-related genes *Runx2*, *Osx*, and *Ocn* in MC3T3-E1 cells after incubation with AdipoRon. The samples were analyzed by quantitative reverse transcription polymerase chain reaction and were normalized to the ß-actin mRNA. The data are the mean from six culture wells (mean ± SEM). **Indicates *P* < 0.01 vs. treatment with AdipoRon alone. **C** Cells were treated with AdipoRon in the presence or absence of AICAR (500 μM). Western blotting was conducted using targeted and β-actin antibodies. Similar results were obtained in four independent experiments

Adiponectin activates AMPK via adiponectin receptors in various types of cells, such as microglia, primary rat osteoblasts, and breast cancer cells [15–17]. In contrast, the decrease in the serum concentration of adiponectin led to the suppression of glucose uptake in the liver and muscles via the inhibition of AMPK in patients with diabetes [18, 19]. Therefore, to clarify whether adiponectin activates osteoblast differentiation in Ti, we examined the impact of AdipoRon, which is an adiponectin receptor type1/type2 activator, on transcriptional activity and the expression of osteoblast differentiation-associated molecules in MC3T3-E1 cells on Ti in the presence or absence of AICAR. AdipoRon (50 µM) gradually increased the transcriptional activity of *Id* (Fig. 5A). The addition of AICAR greatly increased the transcriptional activity.  AdipoRon induced the expression of the *Runx2*, *OSX/Sp7*, and *Ocn* genes in MC3T3-E1 cells on Ti in a time-dependent manner (Fig. 5B). Furthermore, AdipoRon induced the expression of autophagy-associated molecules. AICAR addition provided upregulation for more than 24 h compared with treatment with BMP-2 alone (Fig. 5C; Additional File 1: Fig. S7).

## Discussion

AMPK activation elicited by AICAR promotes the phosphorylation of Smad 1/5/8 in MC3T3-E1 cells, leading to osteoblast differentiation on tissue culture polystyrene dishes (TCPD) [20]. In contrast, BMP-2 phosphorylates Smad1/5, resulting in osteoblast differentiation [21]. In the present experiments, BMP-2 induced the proliferation ratio of MC3T3-E1 cells on a titanium disc in a time-dependent manner, the addition of AICAR, which is an AMPK activator, slightly upregulating them on a titanium disc. Moreover, AICAR also upregulated the BMP-induced *Id* transcriptional activity and the expression of osteogenesis-associated molecules in MC3T3-E1 cells via Smad1/5 phosphorylation on the titanium disc as well as TCPD. Furthermore, BMP-2 in the presence of AICAR induced the expression of their osteoblast differentiation-associated molecules in MC3T3-E1 cells cultured on the titanium disc, to a higher degree than that observed on TCPD (unpublished personal data). We recently reported that AICAR promoted mineralization in MC3T3-E cells on a titanium disc [8]. In line with the results of the present study, those results indicated that AMPK activation dominantly enhanced osteoblast differentiation in MC3T3-E1 cells on a titanium disc as well as previous reported on TCPD, suggesting that AICAR synergistically enhances BMP-induced osteoblast differentiation via the Smad1/5 signaling pathway, at least in part.

Autophagy has been reported to play necessary roles in the homeostasis of bone tissue [22]. Interestingly, numerous undegraded autophagic vacuoles accumulated in the presence of a halted autophagic flux in undifferentiated mesenchymal stem cells [23], suggesting that these accumulated autophagic vacuoles may serve as a source of rapidly produced energy substrates to support MSC differentiation [14, 24]. Osteoblasts are specialized mesenchymal-derived cells that are involved in bone formation; in turn, impaired autophagy in osteoblasts leads to a decreased bone mass [14, 25, 26].

The activation of AMPK, including that observed here, induced autophagy and osteoblast differentiation form stromal/stem cells in bone marrows and periodontal ligaments [6, 13, 27]. In the present experiments, BMP-2 induced the expression of autophagy-associated molecules, such as Beclin-1, BNIP3, and LC3-II, in MC3T3-E1 cells on a titanium disc as well as on TCPD. Furthermore, we found that AICAR, which is an AMPK activator, in the presence of BMP-2 also upregulated the expression of autophagy-associated molecules, especially LC3-II, on the titanium disc. In contrast, 3-MA, which is a PI3K inhibitor, suppressed the expression of autophagy-associated molecules, as well as osteoblast differentiation-associated molecules. Although OIM has been reported to stimulate osteoblast differentiation from mesenchymal stromal cells on TCPD via partially AMPK activation BMP-2 had little effect on AMPK phosphorylation on titanium disc. In present experiments, AMPK activation enhanced osteoblast differentiation from MC3T3-E1 cells on a titanium disc via the activation of autophagy. These results indicated that AMPK activated autophagy, resulting in enhancement of osteoblast differentiation in MC3T3-E1 cells pre-osteoblast on a titanium disc.

Adiponectin is an adipose-derived peptide hormone, exerts anabolic effects to decrease insulin resistance and metabolic disorders by promoting glucose utilization and fatty acid oxidation via AMP-activated protein kinase (AMPK) signaling pathways [28–30]. AMPK activation has been reported to stimulate some downstream signaling pathway, such as Wnt/b-catenin [31], eNOS [32], ERK [33], and autophagy [6] in osteoblast lineages including mesenchymal stem/stromal cells on TCPD, but not titanium disc. In addition, the adiponectin receptor type 1 is expressed in osteoblasts, osteoclasts, and chondrocytes [34].

AdipoRon (APN) has been reported to bind to endogenous adiponectin type 1/type 2 receptors to improve insulin resistance and glucose intolerance [35]. APN promoted new bone formation in patients with diabetes mediated by impaired endochondral ossification-induced delayed bone repair in rodent models of diabetes [16, 36]. The property of APN also induced the mobilization and recruitment of bone-marrow-derived mesenchymal stem cells, to participate in tissue repair [37, 38]. However, little is known about the mechanism of APN in osseointegration in rodents treated with dental implants in vitro. In the present study, we found that APN activated AMPK on titanium disc as well as TCPD and then increased the expression of both osteoblast differentiation and autophagy-associated molecules in osteoblasts, suggesting the enhancing effect of AMPK activators on osteoblast differentiation during osseointegration on titanium discs in vitro.

The percentage of failure in the early phase after dental implantation has recently been reported to be higher than that of the late phase in patients with type II diabetes [39, 40]. Patients with type II diabetes have been reported to exhibit a decrease in the concentration of adiponectin in the serum [41, 42]. In a histological study, the tissues surrounding the dental implant were observed to be immature and less-organized bone in model animals of uncontrolled diabetes [43]. Furthermore, the quality of new bone and osseointegration were significantly reduced, even though insulin-controlled model rats for diabetes [44–46]. Therefore, the lower level of osseointegration in patients with diabetes is a clinically important event that warrants improvement during dental implant therapy. However, little is known whether AMPK activation using its activator enhance earlier and stronger bone-to-implant contact and osseointegration in vivo, even though diabetes model rodents. Further studies are required to clarify these issues.

## Conclusion

In the present study, we found that APN and downstream AMPK signaling enhanced osteoblast differentiation. Furthermore, the upregulation was regulated by autophagy signaling. These results suggest the enhancement of osteoblast differentiation and mineralization in peripheral alveolar bone after implantation, even in patients with diabetes, leading to an early gain of fine osseointegration and implant stability.

### Supplementary Information

**Additional file 1.** Supplemental Material.

## Data Availability

The data sets used and analyzed during the current study are available from the corresponding author on reasonable request.

## Abbreviations

AICAR: 5-Aminoimidazole-4-carboxamide ribonucleotide
AMPK: AMP-activated protein kinase
BMP-2: Bone morphologic protein-2
3-MA: 3-Methhyladnine
APN: AdipoRon
eNOS: Endothelial nitric oxide synthase
ERK: Extracellular signal-regulated kinase

## References

[1] Hardie DG, Ross FA, Hawley SA (2012). AMP-activated protein kinase: a target for drugs both ancient and modern. Chem Biol.

[2] Burkewitz K, Zhang Y, Mair WB (2014). AMPK at the nexus of energetics and aging. Cell Metab.

[3] Wu SB, Wu YT, Wu TP, Wei YH (2014). Role of AMPK-mediated adaptive responses in human cells with mitochondrial dysfunction to oxidative stress. Biochimica et Biophysica Acta (BBA)—General Subjects.

[4] Mu W, Wang Z, Ma C, Jiang Y, Zhang N, Hu K (2018). Metformin promotes the proliferation and differentiation of murine preosteoblast by regulating the expression of sirt6 and oct4. Pharmacol Res.

[5] Ko FC, Kobelski MM, Zhang W, Grenga GM, Martins JS, Demay MB (2021). Phosphate restriction impairs mTORC1 signaling leading to increased bone marrow adipose tissue and decreased bone in growing mice. J Bone Miner Res.

[6] Li Y, Su J, Sun W, Cai L, Deng Z (2018). AMP-activated protein kinase stimulates osteoblast differentiation and mineralization through autophagy induction. Int J Mol Med.

[7] Kanazawa I, Takeno A, Tanaka KI, Notsu M, Sugimoto T (2018). Osteoblast AMP-activated protein kinase regulates glucose metabolism and bone mass in adult mice. Biochem Biophys Res Commun.

[8] Vansana P, Kakura K, Taniguchi Y, Egashira K, Matsuzaki E, Tsutsumi T (2022). The effect of AMP kinase activation on differentiation and maturation of osteoblast cultured on titanium plate. J Dent Sci.

[9] Shetty S, Kusminski CM, Scherer PE (2009). Adiponectin in health and disease: evaluation of adiponectin-targeted drug development strategies. Trends Pharmacol Sci.

[10] Yamauchi T, Kadowaki T (2013). Adiponectin receptor as a key player in healthy longevity and obesity-related diseases. Cell Metab.

[11] Khan MP, Kumar Singh A, Joharapurkar AA, Yadav M, Shree S, Kumar H (2015). Pathophysiological mechanism of bone loss in type 2 diabetes involves inverse regulation of osteoblast function by PGC-1α and skeletal muscle atrogenes: adipoR1 as a potential target for reversing diabetes-induced osteopenia. Diabetes.

[12] Wu YC, Wang WT, Lee SS, Kuo YR, Wang YC, Yen SJ (2019). Glucagon-like peptide-1 receptor agonist attenuates autophagy to ameliorate pulmonary arterial hypertension through Drp1/NOX- and Atg-5/Atg-7/Beclin-1/LC3β pathways. Int J Mol Sci.

[13] Yasunaga M, Kajiya H, Toshimitsu T, Nakashima H, Tamaoki S, Ishikawa H (2019). The early autophagic pathway contributes to osteogenic differentiation of human periodontal ligament stem cells. J Hard Tissue Biol.

[14] Nollet M, Santucci-Darmanin S, Breuil V (2014). Autophagy in osteoblasts is involved in mineralization and bone homeostasis. Autophagy.

[15] Jian M, Kwan JSC, Bunting M, Ng RCL, Chan KH (2019). Adiponectin suppresses amyloid-β oligomer (AβO)-induced inflammatory response of microglia via AdipoR1-AMPK-NF-κB signaling pathway. J Neuroinflamm.

[16] Hu XF, Wang L, Lu YZ, Xiang G, Wu ZX, Yan YB (2017). Adiponectin improves the osteointegration of titanium implant under diabetic conditions by reversing mitochondrial dysfunction via the AMPK pathway in vivo and in vitro. Acta Biomater.

[17] Chung SJ, Nagaraju GP, Nagalingam A, Muniraj N, Kuppusamy P, Walker A (2017). ADIPOQ/adiponectin induces cytotoxic autophagy in breast cancer cells through STK11/LKB1-mediated activation of the AMPK-ULK1 axis. Autophagy.

[18] Li X, Wang Y-X, Shi P, Liu Y-P, Li T, Liu S-Q (2020). Icariin treatment reduces blood glucose levels in type 2 diabetic rats and protects pancreatic function. Exp Ther Med.

[19] Ziqubu K, Dludla PV, Moetlediwa MT, Nyawo TA, Pheiffer C, Jack BU (2023). Disease progression promotes changes in adipose tissue signatures in type 2 diabetic (db/db) mice: The potential pathophysiological role of batokines. Life Sci.

[20] Jang WG, Kim EJ, Lee KN, Son HJ, Koh JT (2011). AMP-activated protein kinase (AMPK) positively regulates osteoblast differentiation via induction of Dlx5-dependent Runx2 expression in MC3T3E1 cells. Biochem Biophys Res Commun.

[21] Zhou X, Xu SN, Yuan ST, Lei X, Sun X, Xing L (2021). Multiple functions of autophagy in vascular calcification. Cell Biosci.

[22] Guan JL, Simon AK, Prescott M, Menendez JA, Liu F, Wang F (2013). Autophagy in stem cells. Autophagy.

[23] Nuschke A, Rodrigues M, Stolz DB (2014). Human mesenchymal stem cells/multipotent stromal cells consume accumulated autophagosomes early in differentiation. Stem Cell Res Ther.

[24] Pierrefite-Carle V, Santucci-Darmanin S, Breuil V, Camuzard O, Carle GF (2015). Autophagy in bone: Self-eating to stay in balance. Ageing Res Rev.

[25] Li H, Li D, Ma Z, Qian Z, Kang X, Jin X (2018). Defective autophagy in osteoblasts induces endoplasmic reticulum stress and causes remarkable bone loss. Autophagy.

[26] Liu F, Fang F, Yuan H, Yang D, Chen Y, Williams L (2013). Suppression of autophagy by FIP200 deletion leads to osteopenia in mice through the inhibition of osteoblast terminal differentiation. J Bone Miner Res.

[27] Veldhuis-Vlug AG, Rosen CJ (2017). Mechanisms of marrow adiposity and its implications for skeletal health. Metabolism.

[28] Yamaguchi T, Sugimoto T (2012). Bone metabolism and fracture risk in type 2 diabetes mellitus. Bonekey Rep.

[29] Okada-Iwabu M, Iwabu M, Yamauchi T, Kadowaki T (2018). Structure and function analysis of adiponectin receptors toward development of novel antidiabetic agents promoting healthy longevity. Endocr J.

[30] Kim AY, Park YJ, Pan X, Shin KC, Kwak SH, Bassas AF, Sallam RM, Park KS, Alfadda AA, Xu A, Kim JB (2015). Obesity-induced DNA hypermethylation of the adiponectin gene mediates insulin resistance. Nat Commun.

[31] Takatani T, Minagawa M, Takatani R, Kinoshita K, Kohno Y (2011). AMP-activated protein kinase attenuates Wnt/β-catenin signaling in human osteoblastic Saos-2 cells. Mol Cell Endocrinol.

[32] Kanazawa I, Yamaguchi T, Yano S, Yamauchi M, Sugimoto T (2009). Activation of AMP kinase and inhibition of Rho kinase induce the mineralization of osteoblastic MC3T3-E1 cells through endothelial NOS and BMP-2 expression. Am J Endocrinol Metab.

[33] Kim JE, Ahn MW, Baek SH, Lee IK, Kim YW, Kim JY, Dan JM, Park SY (2008). AMPK activator, AICAR, inhibits palmitate-induced apoptosis in osteoblast. Bone.

[34] Berner HS, Lyngstadaas SP, Spahr A, Monjo M, Thommesen L, Drevon CA (2004). Adiponectin and its receptors are expressed in bone-forming cells. Bone.

[35] Nicolas S, Rochet N, Gautier N, Chabry J, Pisani DF (2020). The adiponectin receptor agonist AdipoRon normalizes glucose metabolism and prevents obesity but not growth retardation induced by glucocorticoids in young mice. Metabolism.

[36] Huang BoRui, Bi W, Sun Y, Li R, Xingwen Wu, Youcheng Yu (2021). AdipoRon promotes the osseointegration of dental implants in mice with type 2 diabetes mellitus. Front Physiol.

[37] Huang CY, Lee CY, Chen MY, Tsai HC, Hsu HC, Tang CH (2010). Adiponectin increases BMP-2 expression in osteoblasts via AdipoR receptor signaling pathway. J Cell Physiol.

[38] Lin YY, Chen CY, Chuang TY, Lin Y, Liu HY, Mersmann HJ (2014). Adiponectin receptor 1 regulates bone formation and osteoblast differentiation by GSK-3β/β-Catenin signaling in mice. Bone.

[39] de Araújo NM, Maló P, Gonçalves Y, Sabas A, Salvado F (2016). Dental implants in diabetic patients: retrospective cohort study reporting on implant survival and risk indicators for excessive marginal bone loss at 5 years. J Oral Rehabil.

[40] Dubey RK, Gupta DK, Singh AK (2013). Dental implant survival in diabetic patients; review and recommendations. Nat J Maxillofac Surg.

[41] Li S, Shin HJ, Ding EL, Van Dam RM (2009). Adiponectin levels and risk of type 2 diabetes: a systematic review and meta-analysis. JAMA.

[42] Daimon M, Oizumi T, Saitoh T, Kameda W, Hirata A, Yamaguchi H (2003). Decreased serum levels of adiponectin are a risk factor for the progression to type 2 diabetes in the Japanese Population: the Funagata study. Diabetes Care.

[43] Saito N, Mikami R, Mizutani K, Takeda K, Kominato H, Kido D (2022). Impaired dental implant osseointegration in rat with streptozotocin-induced diabetes. J Periodontal Res.

[44] Doll C, Hartwig S, Nack C, Nahles S, Nelson K, Raguse JD (2015). Dramatic course of osteomyelitis in a patient treated with immediately placed dental implants suffering from uncontrolled diabetes: a case report. Eur J Oral Implantol.

[45] De Morais JAND, Trindade-Suedam IK, Pepato MT, Marcantonio E, Wenzel A, Scaf G (2009). Effect of diabetes mellitus and insulin therapy on bone density around osseointegrated dental implants: a digital subtraction radiography study in rats. Clin Oral Implants Res.

[46] Shyng YC, Devlin H, Ou KL (2006). Bone formation around immediately placed oral implants in diabetic rats. Int J Prosthodont.

