# Supplementary material for: AMPK activation enhances osteoblast differentiation on a titanium disc via autophagy
Source: Int J Implant Dent. 2024 Jan 29;10:2. doi: 10.1186/s40729-024-00525-2 (PMC10825085; doi:10.1186/s40729-024-00525-2)
Supplement: Supplementary file 1 — Additional file 1. Supplemental Material. [file 40729_2024_525_MOESM1_ESM.pptx]

## Slide 1
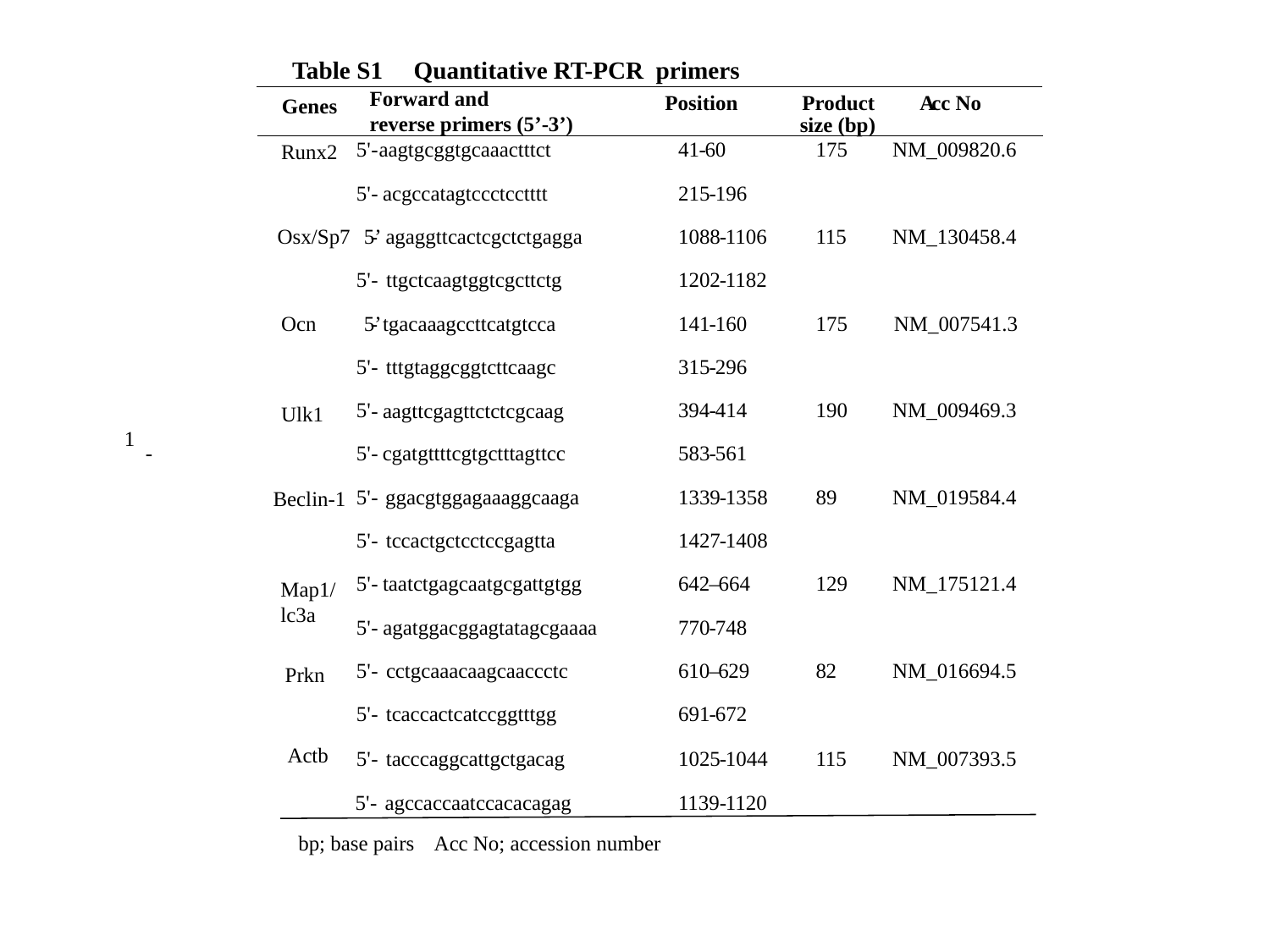

Table S1 Quantitative RT-PCR primers
Forward and
reverse primers (5’-3’)
Position
Product
A
cc No
Genes
size (bp)
5'
-
aagtgcggtgcaaactttct
41
-
60
175
NM_009820.6
Runx2
5'
-
acgccatagtccctcctttt
215
-
196
5’
-
agaggttcactcgctctgagga
1088
-
1106
115
NM_130458.4
Osx/Sp7
5'
-
ttgctcaagtggtcgcttctg
1202
-
1182
Ocn
5’
-
tgacaaagccttcatgtcca
141
-
160
175
NM_007541.3
5'
-
tttgtaggcggtcttcaagc
315
-
296
5'
3
94
-
414
190
NM_009469.3
-
aagttcgagttctctcgcaag
Ulk1
1
-
5'
-
cgatgttttcgtgctttagttcc
583
-
561
5'
-
ggacgtggagaaaggcaaga
1339
-
1358
89
NM_019584.4
Beclin-1
5'
-
tccactgctcctccgagtta
1427
-
1408
5'
-
taatctgagcaatgcgattgtgg
6
42
–
664
129
NM_175121.4
Map1/
lc3a
5'
-
agatggacggagtatagcgaaaa
770
-
748
5'
-
cctgcaaacaagcaaccctc
6
10
–
6
29
8
2
NM_016694.5
Prkn
5'
-
tcaccactcatccggtttgg
691
-
672
Actb
5'
-
tacccaggcattgctgacag
1025
-
1044
115
NM_007393.5
5'
-
agccaccaatccacacagag
1139
-
1120
bp; base pairs Acc No; accession number

## Slide 2
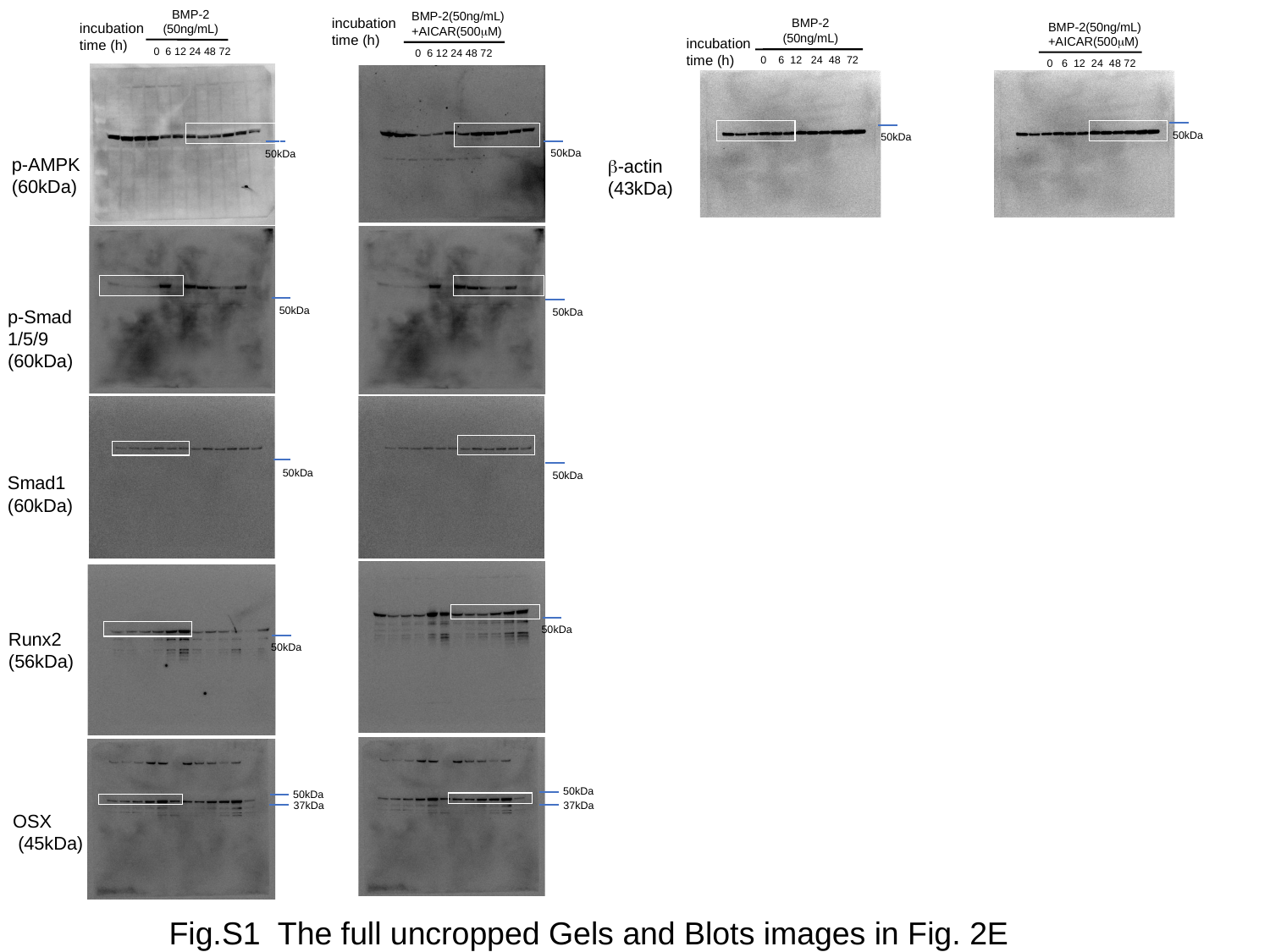

BMP-2
(50ng/mL)
incubation
time (h)
0 6 12 24 48 72
BMP-2(50ng/mL)
+AICAR(500mM)
0 6 12 24 48 72
incubation
time (h)
BMP-2
(50ng/mL)
incubation
time (h)
0 6 12 24 48 72
BMP-2(50ng/mL)
+AICAR(500mM)
0 6 12 24 48 72
50kDa
50kDa
50kDa
50kDa
p-AMPK
(60kDa)
b-actin
(43kDa)
50kDa
p-Smad
1/5/9 (60kDa)
50kDa
50kDa
50kDa
Smad1
(60kDa)
50kDa
Runx2 (56kDa)
50kDa
50kDa
50kDa
 37kDa
 37kDa
OSX
 (45kDa)
Fig.S1 The full uncropped Gels and Blots images in Fig. 2E

## Slide 3
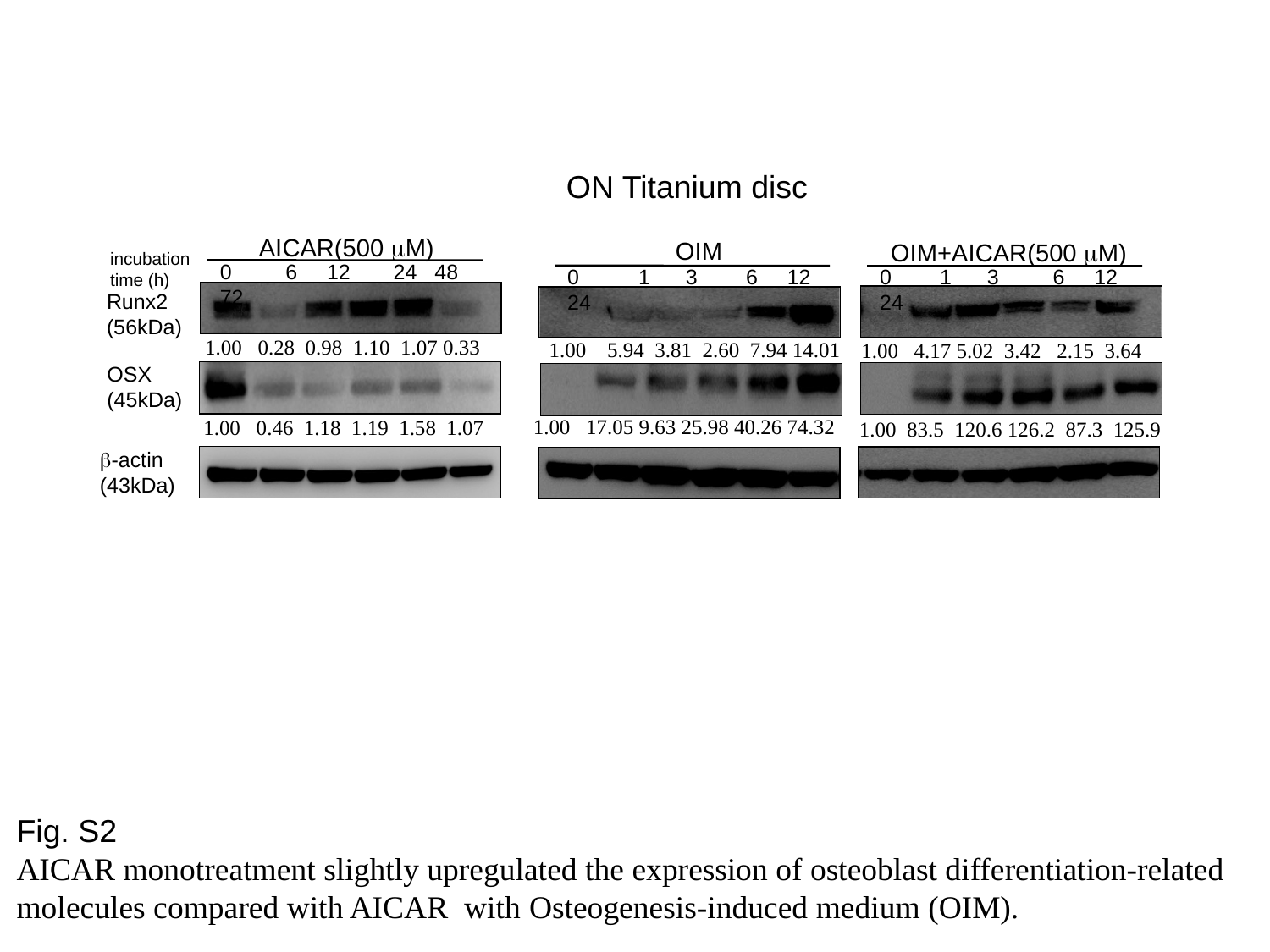

ON Titanium disc
AICAR(500 mM)
OIM
OIM+AICAR(500 mM)
incubation
time (h)
0 　 6 12 　 24 48 72
0 　 1 3 　 6 12 24
0 　 1 3 　 6 12 24
Runx2
(56kDa)
1.00 0.28 0.98 1.10 1.07 0.33
1.00 5.94 3.81 2.60 7.94 14.01
1.00 4.17 5.02 3.42 2.15 3.64
OSX
(45kDa)
1.00 17.05 9.63 25.98 40.26 74.32
1.00 0.46 1.18 1.19 1.58 1.07
1.00 83.5 120.6 126.2 87.3 125.9
b-actin
(43kDa)
Fig. S2
AICAR monotreatment slightly upregulated the expression of osteoblast differentiation-related molecules compared with AICAR with Osteogenesis-induced medium (OIM).

## Slide 4
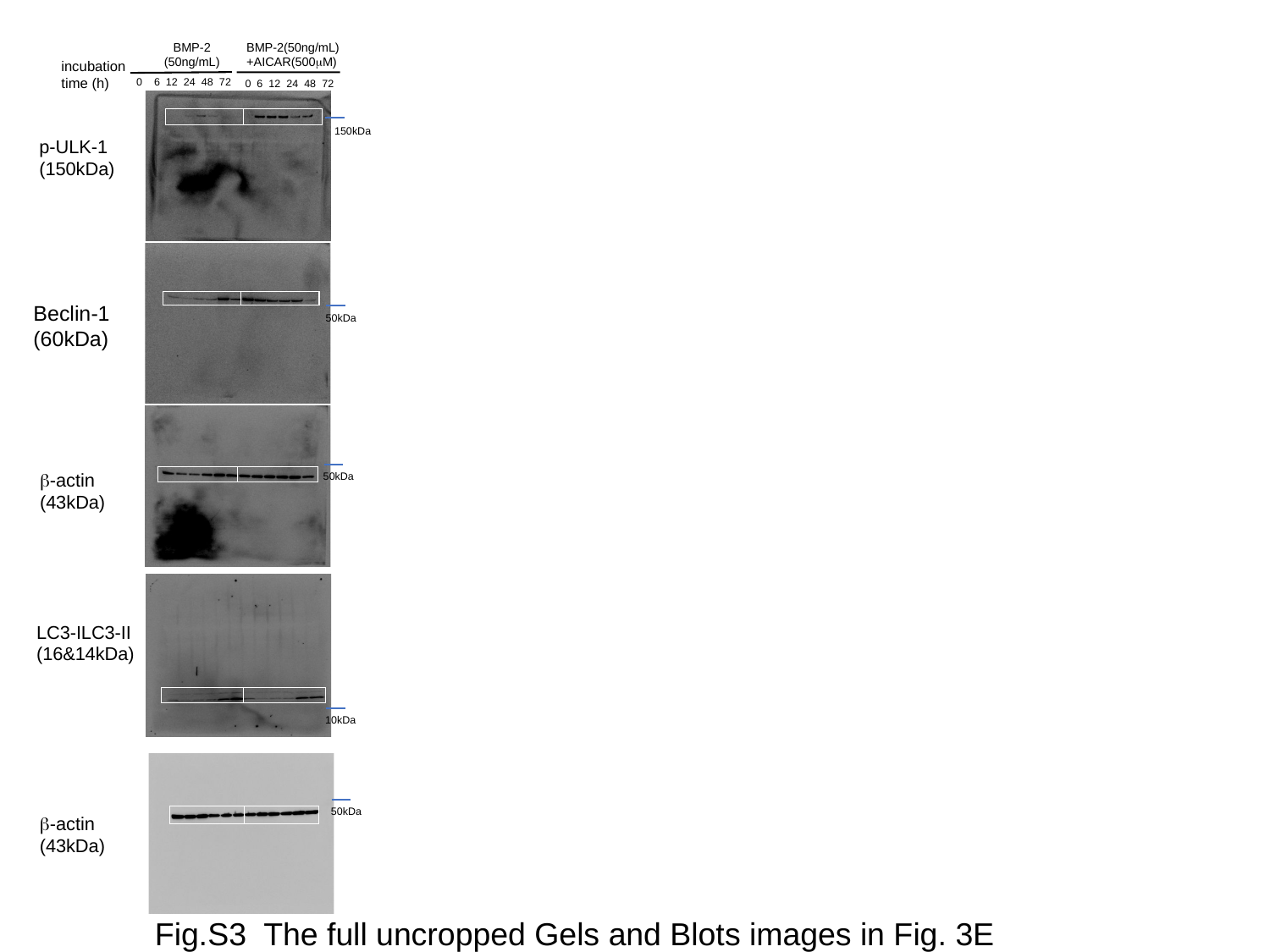

BMP-2
(50ng/mL)
incubation
time (h)
0 6 12 24 48 72
BMP-2(50ng/mL)
+AICAR(500mM)
0 6 12 24 48 72
150kDa
p-ULK-1
(150kDa)
Beclin-1
(60kDa)
50kDa
b-actin
(43kDa)
50kDa
LC3-ILC3-II
(16&14kDa)
10kDa
50kDa
b-actin
(43kDa)
Fig.S3 The full uncropped Gels and Blots images in Fig. 3E

## Slide 5
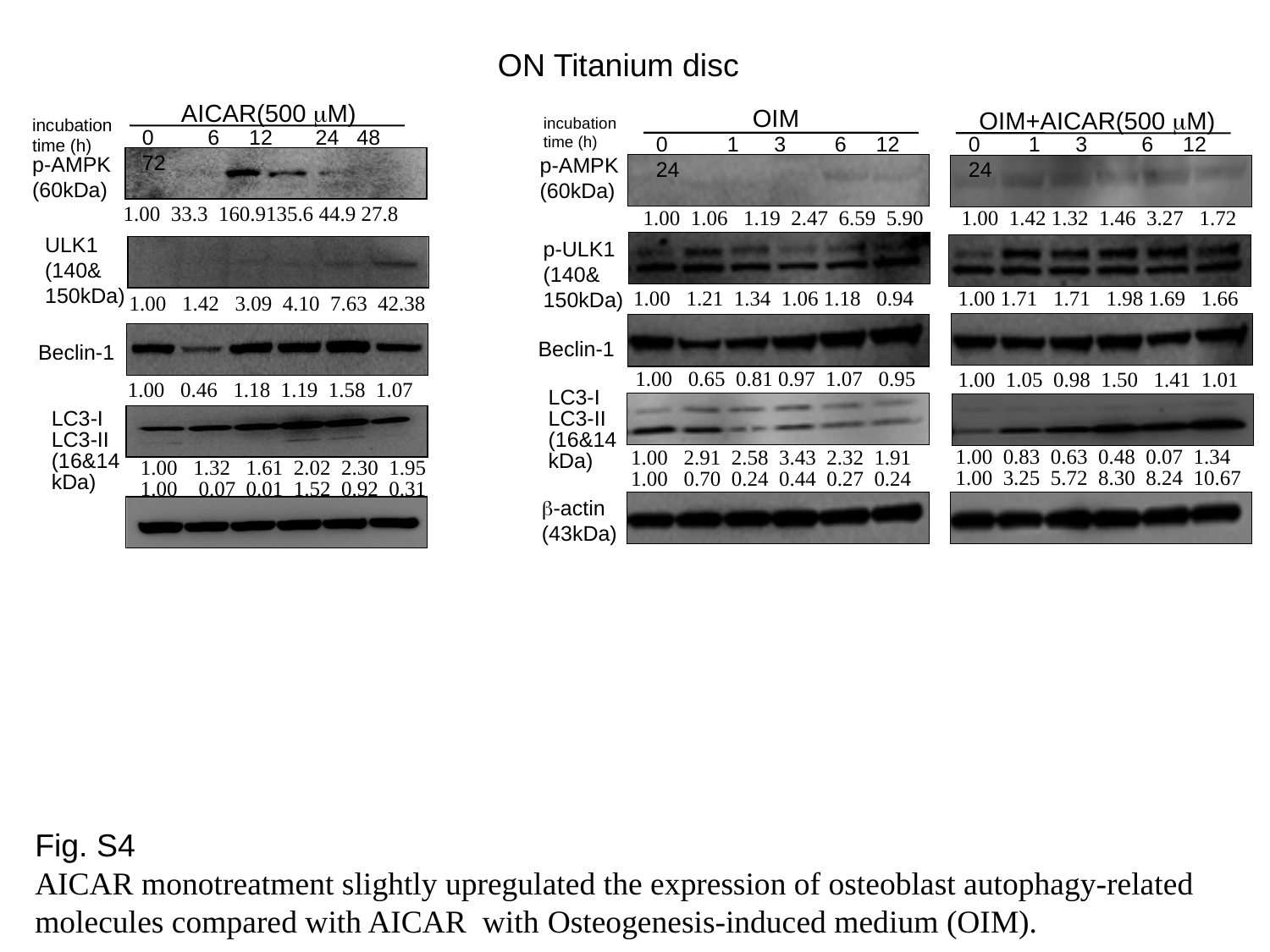

ON Titanium disc
AICAR(500 mM)
OIM
OIM+AICAR(500 mM)
incubation
time (h)
incubation
time (h)
0 　 6 12 　 24 48 72
0 　 1 3 　 6 12 24
0 　 1 3 　 6 12 24
p-AMPK
(60kDa)
p-AMPK
(60kDa)
1.00 33.3 160.9135.6 44.9 27.8
1.00 1.06 1.19 2.47 6.59 5.90
1.00 1.42 1.32 1.46 3.27 1.72
ULK1
(140&
150kDa)
p-ULK1
(140&
150kDa)
1.00 1.71 1.71 1.98 1.69 1.66
1.00 1.21 1.34 1.06 1.18 0.94
1.00 1.42 3.09 4.10 7.63 42.38
Beclin-1
Beclin-1
1.00 0.65 0.81 0.97 1.07 0.95
1.00 1.05 0.98 1.50 1.41 1.01
1.00 0.46 1.18 1.19 1.58 1.07
LC3-I
LC3-II
(16&14
kDa)
LC3-I
LC3-II
(16&14
kDa)
1.00 0.83 0.63 0.48 0.07 1.34
1.00 3.25 5.72 8.30 8.24 10.67
1.00 2.91 2.58 3.43 2.32 1.91
1.00 0.70 0.24 0.44 0.27 0.24
1.00 1.32 1.61 2.02 2.30 1.95
1.00 0.07 0.01 1.52 0.92 0.31
b-actin
(43kDa)
Fig. S4
AICAR monotreatment slightly upregulated the expression of osteoblast autophagy-related molecules compared with AICAR with Osteogenesis-induced medium (OIM).

## Slide 6
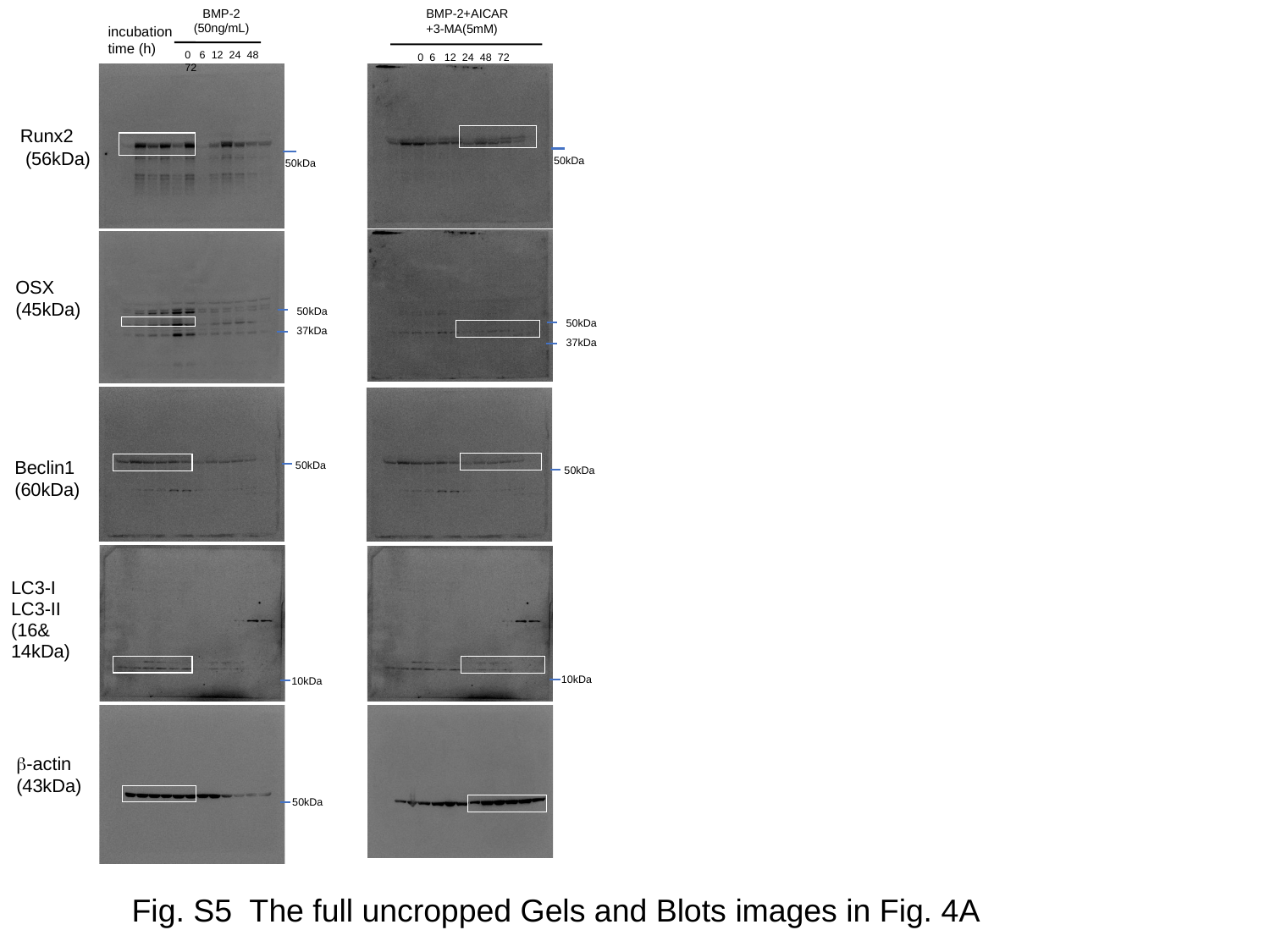

BMP-2+AICAR
+3-MA(5mM)
BMP-2
(50ng/mL)
incubation
time (h)
0 6 12 24 48 72
0 6 12 24 48 72
Runx2
 (56kDa)
50kDa
50kDa
OSX
(45kDa)
50kDa
50kDa
 37kDa
 37kDa
Beclin1
(60kDa)
50kDa
50kDa
LC3-I
LC3-II
(16&
14kDa)
10kDa
10kDa
b-actin
(43kDa)
50kDa
Fig. S5 The full uncropped Gels and Blots images in Fig. 4A

## Slide 7
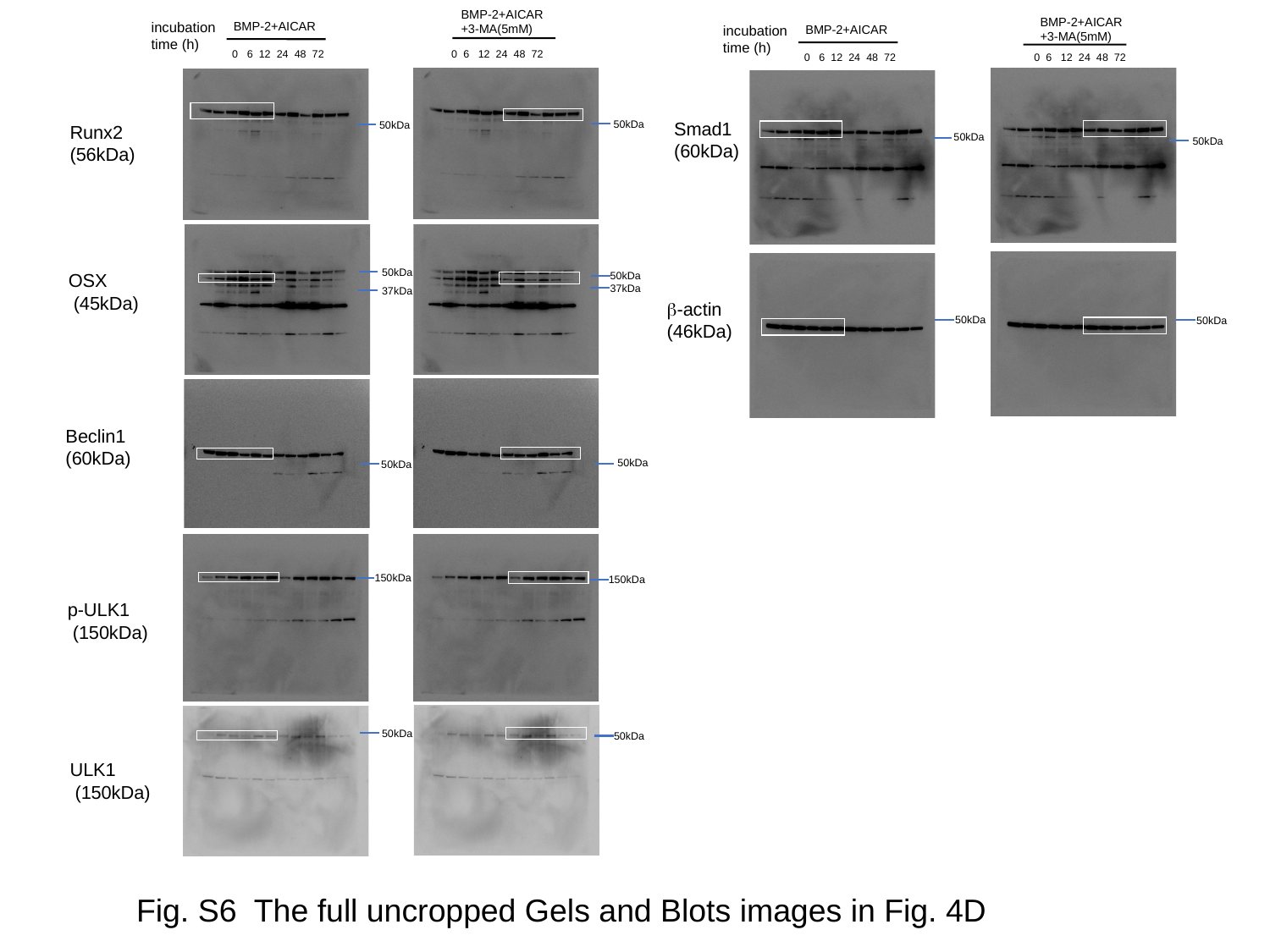

BMP-2+AICAR
+3-MA(5mM)
BMP-2+AICAR
+3-MA(5mM)
incubation
time (h)
BMP-2+AICAR
0 6 12 24 48 72
incubation
time (h)
BMP-2+AICAR
0 6 12 24 48 72
0 6 12 24 48 72
0 6 12 24 48 72
Smad1
(60kDa)
50kDa
50kDa
Runx2
(56kDa)
50kDa
50kDa
50kDa
OSX
 (45kDa)
50kDa
37kDa
37kDa
b-actin
(46kDa)
50kDa
50kDa
Beclin1
(60kDa)
50kDa
50kDa
150kDa
150kDa
p-ULK1
 (150kDa)
50kDa
50kDa
ULK1
 (150kDa)
Fig. S6 The full uncropped Gels and Blots images in Fig. 4D

## Slide 8
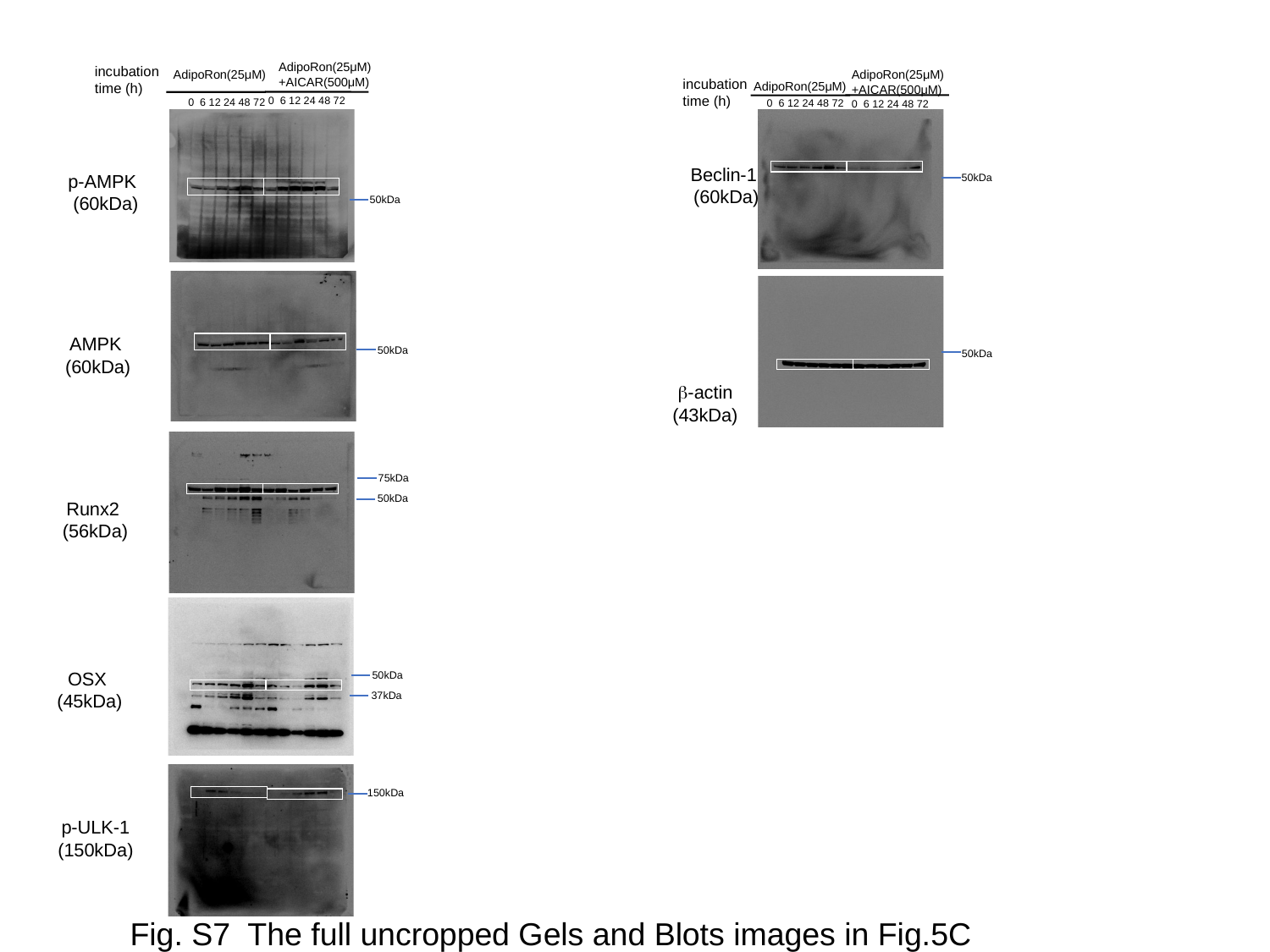

AdipoRon(25μM)
+AICAR(500μM)
incubation
time (h)
AdipoRon(25μM)
 0 6 12 24 48 72
AdipoRon(25μM)
+AICAR(500μM)
incubation
time (h)
AdipoRon(25μM)
0 6 12 24 48 72
0 6 12 24 48 72
0 6 12 24 48 72
Beclin-1
 (60kDa)
p-AMPK
 (60kDa)
50kDa
50kDa
b-actin (43kDa)
AMPK
 (60kDa)
50kDa
50kDa
75kDa
50kDa
Runx2
 (56kDa)
OSX
(45kDa)
50kDa
37kDa
150kDa
p-ULK-1 (150kDa)
Fig. S7 The full uncropped Gels and Blots images in Fig.5C
